# Building Public Health Capacity through Organizational Change in the Sport System: A Multiple-Case Study within Australian Gymnastics

**DOI:** 10.3390/ijerph18136726

**Published:** 2021-06-22

**Authors:** Amy Carrad, Anne-Maree Parrish, Heather Yeatman

**Affiliations:** School of Health and Society, Faculty of the Arts, Social Sciences and Humanities, University of Wollongong, Northfields Ave, Wollongong, NSW 2522, Australia; aparrish@uow.edu.au (A.-M.P.); hyeatman@uow.edu.au (H.Y.)

**Keywords:** health promotion, sport, settings-based approach, organizational change, gymnastics

## Abstract

Sports clubs increasingly are settings for health promotion initiatives. This study explored organizational change processes and perceived facilitators and barriers relevant to implementing a health promotion initiative within gymnastics settings in New South Wales (NSW), Australia. A multiple-case design investigated the experiences of the state association (Gymnastics NSW) and five clubs from one region of NSW in a participatory Health-Promoting Gymnastics Clubs (HPGC) program. The program aimed to build the capacity of Gymnastics NSW to support affiliated clubs to become health-promoting settings. Interviews with organizational representatives explored their experiences of the program and identified factors that enabled or inhibited program adoption, implementation and sustainability. Facilitators and barriers identified included leadership and champions; organizational capacity and culture; priorities and timing; and characteristics of the HPGC framework. This multi-level, organizational change intervention demonstrated potential to create health-promoting gymnastics settings. Tailoring strategies in diverse club contexts required involvement of organizational leaders in program development and action planning. Despite positive impacts, pre-existing organizational culture inhibited integration of health promotion as a core value. Sustained organizational change may result from professional regulatory requirements (e.g., accreditation and affiliation), and policy directives and funding (for organizational change, not program delivery) from relevant government departments.

## 1. Introduction

The World Health Organization (WHO), public health professionals and researchers have long advocated for a settings-based approach to promote the health of individuals, communities and populations. As described in the *Ottawa Charter for Health Promotion (‘Ottawa Charter’)*, settings are the places where people “learn, work, play and love” [1]. Settings-based health promotion recognizes that environmental, economic, political, social and cultural determinants shape health and illness [2] and that it is necessary to look beyond individual factors alone when striving to generate and maintain wellbeing. While health promotion initiatives have taken place in schools, workplaces, prisons and towns, there has been recent interest in organized sport as a setting within which health becomes an innate part of culture, structures, processes and routine operations [3,4,5]. 

Participation in organized sport carries with it various physical, mental and social benefits for individuals [6,7] and enhanced social capital for communities [8]. However, some research has shown that participants of organized sport can have negative behaviors, such as alcohol consumption and intake of sugary and fatty foods, comparable to or worse than people who do not participate in sport [9,10]. Additionally, sport can lead to environmental exposures that contradict a healthy lifestyle, such as the normalized consumption and use of tobacco [11], alcohol, junk food and gambling [12,13] through sponsorship, advertising, rituals and product sales. The environmental exposures extend not only to players but also to others who are involved in sport, such as players’ families, coaches, spectators and volunteers [14].

High rates of participation in organized sport and the structured organizational nature of the Australian sports sector make it a promising system within which to incorporate health promotion. In Australia, organized sport is a popular recreation activity, particularly among children, with approximately 1.7 million (60%) children aged 5–14 years having participated in at least one organized sport in the past year [15]. An estimated 3.8 million Australians over the age of 15 years play a sport [16], approximately 96,000 people are employed by the sector [17], and over 2.3 million participate through volunteering [18]. A common hierarchy for the delivery of sport in Australia is a tiered system [19]. National administration is conducted by the Australian Sports Commission and Federal Government, and national sporting organizations (NSOs) promote elite competition and grassroots participation. State sporting organizations (SSOs) oversee state-based responsibilities such as implementing policy frameworks and requirements for clubs. At the most proximal level, local government and local sports clubs conduct day-to-day tasks to ensure people can participate in sport [19]. 

Previous sports-based health promotion research has included non-empirical work, descriptive studies and evaluations of interventions. At a conceptual level, researchers have produced guiding frameworks and standards for health-promoting sports clubs (HPSC) [20,21,22] and applied Whitelaw et al.’s [23] health-promoting settings typology to sport-based initiatives [24]. Contextualized within sport, Kokko, Green and Kannas [24] described three levels of health promotion activities: passive, club-based education and club-society development. Passive approaches entail the club being used as a conduit for health promotion professionals and researchers to reach individuals to deliver health education programs; for example, screening for sexually transmitted infections through football club outreach programs [25]. Club-based education approaches engage athletes, coaches and administrators in conducting the health promotion activities; for example, provision of healthy snacks to athletes by coaches [26]. Lastly, club-society development approaches feature system-wide changes to organizational culture, structure and ethos that result in health promotion being included in core business and thus in policy and routine practice. Some examples of this latter approach include: initiatives from VicHealth in the Australian state of Victoria that use a health promotion sponsorship model [27,28,29,30,31]; the Australia-wide Good Sports program [32,33,34]; Sweden’s Goal without Alcohol program [35]; and the European Healthy Stadia Network [36].

Existing research on HPSCs has largely been cross-sectional and descriptive in nature, with few controlled interventions reported [3,5,37,38]. Internationally, research evaluating the implementation of HPSC interventions has predominantly been expert driven, conducted within male-dominated team sports, addressed a singular health topic (such as healthy eating), and has not embodied a club-society development approach to health-promoting settings [3]. Few studies have conducted an in-depth exploration of the cultural and structural context of the organization or sport type as a whole, instead focusing on environmental or health outcomes (e.g., presence of a policy or healthier food purchasing patterns). 

Evaluations of the VicHealth sponsorship model included exploration of organizational factors that enabled or inhibited health promotion; however, the model’s limitations were that it took a top-down approach whereby sponsorships were received by the SSO and clubs were not involved in the process of negotiating sponsorship contracts yet were expected to implement changes at the local level [31]. Challenges to effecting structural change included the health messages being determined by VicHealth and not the clubs, inadequate communication between the state and local level of sports, and the disconnect of the funding body [31]. Top-down approaches are likely to result in a lack of community ownership and participatory decision making, fundamental to building organizational capacity and improving the opportunities for sustainability [39].

HPSC projects need to embrace the three key characteristics of health-promoting settings described by Dooris [40]: ecological models, whole-of-systems approaches and organizational change processes. Particularly within the hierarchical structure of sport in Australia, it is important to investigate the whole system with consideration of how the various components of the system are interrelated.

The aim of this research was to inform the feasibility of creating health-promoting sport organizations through exploring the processes and perceived facilitators and barriers to organizational change within an SSO and clubs participating in the Health-Promoting Gymnastics Club (HPGC) program. 

## 2. Theoretical Background

### 2.1. Organizational Change Theory

Organizational change is a complex field that some have argued is fundamental to any true health-promoting settings initiative [41,42]. This study adopted the following definition of organizational change: ‘an organization’s transition from a past to a new or future state, which may involve adaptations to underlying ways of thinking, being and operating’ [43]. Throughout this process, it is the organizational system and structures and groups within it that are the primary targets of change, rather than individuals [44,45]. In relation to health promotion, organizational change ultimately seeks to embed health promotion within the core business, values and culture of organizations and settings [46].

The literature regarding organizational change theory is extensive, including perspectives describing different types, process models and factors that enable or inhibit change. Organizational change types have been characterized on ‘dimensions’. Examples of dimensions include: organization-wide vs. subsystem change; transformational vs. incremental change; remedial vs. developmental change; and proactive vs. reactive change [47]. Another characteristic, ‘mode’, includes the depth within the organization at which the change occurs, such as changes to observable artefacts or more fundamental transformations to organizational culture [48]. ‘Level’ refers to whether change occurs within or across organizations [49]. Finally, duration is the time over which change takes place [44]. Recognizing and exploring the possible types of change anticipated or expected may be beneficial for the effective negotiation and management of change processes [44].

Much of the field of organizational change has been concerned with understanding and describing processes or patterns of change by mapping the possible paths that change may take. This may assist organizations to manage the change process and thus enhance the likelihood of achieving the desired strategic, structural and cultural outcomes [44]. The change process has often been thought of as linear [45,50,51], whereby organizations transition from one state to another along a pathway where one stage neatly follows the preceding stage and with a finite start and end. Alternative process models describe cyclical processes [52,53], which similarly propose a stage-based approach while also recognizing the ongoing nature of change. 

Complexity theory is a third way of explaining organizational change processes [54]. It acknowledges the often unpredictable, dynamic and discontinuous nature of transformation. Complexity theory posits that organizational change occurs in a non-linear fashion due, in part, to the large number of interdependent, interacting elements within the organizational system that pose challenges to predicting and planning change [55,56]. Thus, complex or adaptable models of organizational change processes [54] have potential utility in better enabling change practitioners to design, implement and evaluate organizational change projects in sports settings.

### 2.2. Facilitators and Barriers to Organizational Change in the Context of Health-Promoting Sport Clubs

Previous studies on organizational change, including those concerned with sport settings, have explored factors that facilitate, inhibit or prevent change from taking place when introducing an innovation (i.e., health promotion within sports) [27,57,58]. These factors can be part of the organization, the innovation itself, and the surrounding context. Identifying and understanding these factors can be beneficial for designing and implementing organizational change initiatives to enhance the likelihood of achieving the desired outcomes [27].

There have been multiple facilitators of organizational change identified. Facilitators include: the presence of a program champion [29,35,57,59,60]; supportive and authoritative leadership [27,61,62,63]; adequate and appropriate technical support both in the form of health literacy and organizational change processes (e.g., policy templates, professional development for club officials, one-on-one consultation between clubs and health promotion experts, and achievement frameworks) [57,64,65]; participatory approaches [66]; innovation compatibility and adaptability [22,28,31,35]; and examples of legislation or health promotion policies in other settings [32,57,63,67]. 

Generally, the absence of any of the facilitators listed above may constitute a barrier to an organizational change approach for health promotion in sport settings. Additional barriers raised in previous studies include limited health promotion knowledge of stakeholders [60,63,68,69,70,71]; lack of organizational priority for health promotion [68,72,73]; actual and perceived financial shortages [29]; and poor communication between the tiers of sport and to external project partners [30,63]. 

While some sport-based health promotion studies have drawn explicitly on organizational change theory to guide and analyze implementation [27,57], none have done so in the context of a female-dominated sport or with a specific focus on the interaction between the SSO and local clubs. This research sought to address this gap through post-intervention exploration with participants of a multi-level, participatory health promotion intervention within the sport of gymnastics in New South Wales (NSW), Australia.

## 3. Materials and Methods

The study used a multiple-case design to investigate organizational change within the context of a participatory health promotion intervention. It explored the experiences of representatives from an Australian Gymnastics SSO and affiliated local-level clubs. The case-study approach gave detailed attention to the contextual conditions of each participating organization [74] and answered ‘how?’ and ‘why?’ organizational change occurred in light of the contextual factors pertinent to each case [75]. The multiple-case-study approach enabled investigation of different perspectives of organizational change within the nested organizational units (state peak body and local clubs) of (gymnastics) sport in Australia. 

### 3.1. Research Settings

The cases of study for this research were Gymnastics NSW, the SSO for gymnastics in NSW, Australia, and five purposively sampled local-level gymnastics clubs affiliated with the SSO. Gymnastics NSW exists under the National Sporting Organization, Gymnastics Australia, but retains the organizational flexibility to determine its own strategic direction in delivering the sport. Its key activities include acting as a customer service organization to approximately 190 clubs by supporting clubs to operate successful businesses and conducting activities to increase participation in the sport. At the time of the study in 2016, there were 56,477 registered gymnasts in the state [76]. 

All clubs (n = 17) in one region of the state were invited, via email, to participate in the HPGC program. Of these, five clubs provided consent and engaged in the initial action planning process, described below. Recruitment of clubs from the same geographic region occurred at the request of Gymnastics NSW, to facilitate delivery of the health promotion program in line with the Development Department’s existing model of support to clubs. Three of the five clubs were privately-owned businesses and offered gymnastics programs only (hereafter GOC, gymnastics-only club). The remaining two, one a council-operated leisure center and one a Police Citizens Youth Club (PCYC), were multi-sport centers (hereafter MSC), offering activities such as fitness classes, swimming, boxing and weights training in addition to gymnastics. These clubs varied based on: the number of registered gymnasts; physical characteristics; gymnastics programs offered; number of staff, coaches and volunteers; communication channels used; and existing partnerships with external organizations (Table 1). Each of the clubs had at least 100 registered gymnasts at the time of the study, with the largest club being GOCc, which had at least 300 members. The two MSCs had a manager who oversaw the entire center, additional administrative staff and approximately 10 coaches employed on a casual basis. In contrast, the GOCs had at most two staff members responsible for administrative matters, and these staff were also heavily involved with coaching. Examining gymnastics clubs with varied contextual backgrounds provided the opportunity to analyze diverse experiences of the HPGC program, described below, thus demonstrating similarities and differences in contexts and processes [77,78].

Gymnastics NSW provided a letter of support, via email, that indicated the organization’s willingness and approval to participate in the research. Informed, written consent was given by all respondents prior to their participation in the study. The University of Wollongong’s Human Research Ethics Committee approved the protocol (HE15/328; HE16/085).

### 3.2. Procedure

The importance of the definition of health promotion given in the *Ottawa Charter* (“the process of enabling people to increase control over, and to improve their health”) [1] is that health promotion is a means to an end and not just an outcome in itself. The principles of health promotion, such as enabling [79,80], empowering [79,81,82], capacity building [80,83] and facilitating participation [80,81], are highlighted as necessary components. These principles underpinned the formation of the intervention at the heart of this study. The HPGC program was a participatory health promotion initiative designed through collaboration between the first author and Gymnastics NSW. The characteristics of the research settings, organizational change concepts and health promotion principles from the existing literature informed the development and implementation of the intervention. 

Gymnastics NSW’s Development Department strengthened clubs through improving business operations, programs and workforce capacity. Gymnastics NSW identified the Club Development portfolio within this department as an appropriate avenue through which to implement the HPGC program due to its pre-existing responsibilities and operations. The Club Development Officer’s role was to build rapport with clubs and provide tailored guidance on matters such as quality assurance, risk management, insurance and facility development.

Gymnastics NSW’s primary responsibility in the project was to support clubs to implement and deliver the HPGC program. The role of the lead researcher [A.C.] was predominantly to act as a support and facilitator for organizational change planning and implementation and as data collector and evaluator, rather than providing direct assistance to clubs when implementing health promotion activities. The researcher and Gymnastics NSW recognized the delineation of roles during the process of delivering the program.

A major element of the program delivery model was that each participating club created a health promotion action plan that considered their unique context and resources [84]. Between May and June 2016, separate face-to-face meetings between a club representative and Gymnastics NSW staff member allowed each club to discuss health promotion priorities and areas of interest and to generate initial strategies for the HPGC program. Action plans identified the overall health promotion goals of the club and the specific objectives, strategies, activities, timelines, personnel and resources that would contribute to achieving the goals.

Following the initial action planning meeting, clubs began implementing their proposed activities with varied levels of assistance from Gymnastics NSW. Where possible, they did so using the staffing, knowledge and financial assets already available within their club. In other cases, the priorities and actions identified required health promotion resources (e.g., posters, information and existing online programs), which were provided through the tiered support structure of the first author and Gymnastics NSW. Gymnastics NSW connected clubs to these resources in the first instance, but when they themselves needed assistance finding appropriate resources, they contacted the first author. Clubs did not receive direct financial assistance from Gymnastics NSW, but the state organization assisted one to apply for the Make Healthy Normal [85] grant, although they were unsuccessful. Another of the clubs was awarded funding under the Make Healthy Normal [85] grant, having sought assistance in their application from the local health district health promotion unit.

Throughout the duration of the HPGC program (June 2016 to March 2017), clubs implemented a range of activities targeting the areas of healthy eating and nutrition, food environments, mental wellbeing, injury prevention, smoking cessation for staff and physical activity. The approaches taken included environmental change (e.g., removing vending machines selling unhealthy foods and drinks) and professional development for staff (e.g., improving health literacy) (Table 1).

### 3.3. Data Collection

Participant numbers were determined by the number of eligible, consenting participants and number of organizations involved in the HPGC program, rather than by an aim to achieve data saturation. One-on-one post-intervention semi-structured interviews were the primary source of data for the study. Semi-structured question guides based on elements of organizational change theory and findings of prior HPSC research (for example, Casey et al. 2009 [29]; Geidne et al. 2013 [35]; Casey et al. 2012 [57]; Olstad et al. 2012 [60]) [29] explored participants’ experiences and perceptions of the HPGC program, including facilitators and barriers to participation and implementation (Supplementary File S1). The guide was piloted with an academic colleague with expertise in the field of HPSCs and involvement in sport as an administrator, and revisions were made based on the feedback given. The first author [A.C.] conducted the interviews, which lasted between 30 and 70 min, at the respective workplaces of each participant. The researcher responded to issues raised by participants by probing for further explanation and seeking clarification when necessary. Interviews were audio recorded and transcribed verbatim. 

### 3.4. Data Analysis

Interviews were analyzed using thematic analysis [86,87]. Broad deductive categories were determined by the aims of the evaluation and the interview guide [88]. While reviewing each transcript, the researcher made initial notes on data content in Microsoft Word (Microsoft Corp., Redmond, WA, USA). Sections of the transcripts were then labelled with a code [86]. Following coding of all transcripts, the researcher grouped the codes into common themes in Microsoft Excel (Microsoft Corp., Redmond, WA, USA). These themes were reviewed to ensure data within each theme were coherent and each theme was distinct from the others [86]. The first author independently and solely performed the coding process. Peer debriefs with the co-authors discussed possible variations in the interpretation of participants’ responses and clarified meanings.

Trustworthiness, or reliability, of the research was achieved through a number of strategies to address credibility, transferability, dependability and confirmability [89]. Regarding credibility, the first author was familiar with gymnastics due to their prolonged involvement with the sport as an athlete and coach and was thus able to establish rapport more easily with respondents [89]. Using research methods appropriate to the research aims and conducting peer debriefing during data analysis also contributed to establishing credibility. Using the description of the research settings provided above, readers can determine whether the study findings may be transferable to other settings of interest. Illustrative quotations are used in the presentation of the results to exemplify the themes identified through the coding process. Field notes regarding both methodological components and the joint emic–etic approach [90] were kept by the researcher to aid the process of reflexivity [91]. Additionally, during the interviews, participants were asked to comment on the perceived impact of the researcher on the conduct of the HPGC program, and this was considered when analyzing the data.

## 4. Results

Post-intervention interviews with participants from clubs (n = 5 in senior management roles and/or club owner) and Gymnastics NSW (n = 2 from the Development Department) revealed several facilitators and barriers to adopting, implementing and sustaining health-promoting activities in these organizations. These included factors relevant to the individual respondents, organizational components and the project framework. Particular factors were relevant in some organizations but not in others and could be facilitators, barriers or both (Table 2). Leadership, strategic capacity, human capacity and organizational priorities and timing were factors discussed by representatives from all participating organizations. The representative from Club-MSCa spoke using a negative framing for each factor. In contrast, Club-GOCa and Club-MSCb’s representatives used generally more positive framing when discussing the factors that influenced their implementation of the HPGC program. Club-GOCb, Club-GOCc and Gymnastics NSW staff were balanced between positive and negative framing.

### 4.1. Organizational Leadership and Champions

Leaders of the different organizations fundamentally influenced the outcomes of the HPGC program for their respective organizations. Their attitudes toward, interest in and understanding of health promotion shaped the adoption and implementation of activities during the intervention. Strong leadership from within Gymnastics NSW led to the immediate adoption of the HPGC program concept. The Development Manager demonstrated an understanding of the holistic nature of health promotion and was eager to show Gymnastics NSW was “doing something broader than just physical participation.” The positive attitude of the Development Manager toward health promotion was a significant facilitator in program adoption, stating, “I was there from the start because it is something I believe in…”. Strategically, the Development Manager and Club Development Officer worked together to initiate conversations with the participating clubs and utilized the usual tasks conducted within the Club Development portfolio to provide follow-up support to clubs throughout the intervention. The positive attitudes and personal health promotion interests of Gymnastics NSW staff influenced the nature of the program. 

At club level, one person assumed responsibility for the decision to participate in the project and determined the level of involvement. This was due to the largely hierarchical organizational structures of participating clubs and the organizations having one person in a central leadership role. With the exception of Club-MSCa, this resulted in successful adoption of the HPGC concept using varying levels and styles of implementation. For Club-MSCa, the leader’s perceptions of the (low) importance of health promotion in relation to the core business of the club and the relatively low contribution of gymnastics to the broader center within which the club operated proved to be a barrier to participating in the program. Further, this participant acknowledged that in order to engage with the HPGC program, the gymnastics section would “probably need a couple of leaders”; however, such people were not present at the time of the study due to the transient nature of their casual staff.

The personal attributes of the leaders from Club-GOCa and Club-MSCb and their strong support for participation in the HPGC program resulted in these clubs implementing an array of strategies across their respective settings that involved and impacted staff and members. These two leaders had knowledge and understanding of health promotion, enabling them to generate ideas for the project with no or minimal support from Gymnastics NSW. Unprompted mention of social determinants, evidence-based programs and bottom-up approaches by the leader from Club-MSCb demonstrated her informed understanding of health promotion. This comprehensive understanding was reflected in the participatory process to involve staff in the implementation process, and the subsequent ownership staff took over elements of the program. 

The leader from Club-GOCb was interested in intervening in gymnasts’ mental wellbeing to benefit performance outcomes at competitions. This focus on competition and the leader’s perception the club’s opportunities for health promotion were limited because “we were already doing it” formed a barrier to implementing health promotion more holistically. The leader from Club-GOCc conceptualized the nature of health in sport as comprising physical activity and good nutrition rather than a holistic perception of health, and this resulted in the club’s only activity being the provision of free fruit for members when at the club. This participant indicated satisfaction with this achievement and believed it was a sufficient contribution, “We thought that what we were doing with the fruit was enough for us” (Participant-GOCc).

### 4.2. Organizational Capacity 

In the context of this project, organizational capacity comprised the three aspects of strategic (organizational goals and structure), human (staff time and workload) and financial capacity.

#### 4.2.1. Strategic Capacity

The strategic capacity of Gymnastics NSW was an asset to the HPGC program. The Development Manager reported that one of the goals or purposes of the Development Department was to embrace innovation. The invitation to participate in the project was one such opportunity. The nature of support (technical and informational) provided to clubs by Gymnastics NSW mirrored what was offered under previous club development opportunities; thus, the staff members felt comfortable engaging with clubs through the HPGC program delivery framework.

For most club representatives, the HPGC program did not align with the strategic goals or core business of the club, and they reported health promotion as an optional add-on to their usual business. These clubs did not prioritize health promotion in organizational planning, nor did the project provide any impetus for them to review their organizational mission or vision. 

Participants-GOCa and GOCb reported small positive impacts of the program, including perceptions the organizational culture had “slightly changed for the better towards health promotion” (Participant-GOCa), and it had caused the club staff to “think about what we do here and how we do that” (Participant-GOCb). By contrast, Participant-MSCb described health promotion as being integral to the goals of the organization in supporting the physical and social wellbeing of members and the local community, expressing, “We have a moral duty of care to support these families and give them the best start in life”. 

Organizational structure also influenced the strategic capacity of participating organizations. All clubs had hierarchical organizational structures with a central management that, for most, resulted in a lack of willingness to involve or delegate to others—such as other staff and parents—within the club setting. The organizational structures of clubs-MSCa, MSCb and GOCc were barriers to the autonomy of respective project leaders to create and implement consistent change within their settings because for these clubs, gymnastics was one segment of the broader center or the club, over which they felt they had limited influence.

#### 4.2.2. Human Capacity

Human capacity, comprising available time and existing workload of project participants, influenced the HPGC program. Gymnastics NSW staff identified this factor as a major barrier to implementation and perceived sustainability of the project. These participants reported the human resources and time required to implement (action planning and follow up with clubs) the project adequately exceeded their initial expectations and personal capacity. Both Gymnastics NSW participants perceived that an additional staff member dedicated to the health promotion portfolio would allow this person to take ownership of the program and likely lead to better engagement with clubs and thus project outcomes while simultaneously allowing existing staff to maintain their workload.

All club participants discussed the concept of staff time during the post-intervention interviews. Overall, they expressed having limited time and that this restricted their participation in the HPGC program, and their ability to balance club administration, coaching and their personal commitments such as to family. The Club Development Officer was aware of the impact of limited human capacity within clubs on the project. She acknowledged clubs in the program “tended to be on the smaller side” and “the person that we approached was the head coach and was running the business and so quite time poor.” The Club Development Officer also reported clubs provided feedback during the project, indicating concerns regarding the time demands of the HPGC program.

#### 4.2.3. Financial Capacity

Financial capacity was not a barrier to implementing health promotion activities; however, many did report fiscal issues as being indirect barriers to the sustainability of their initiatives and club finances. For all organizations, there was no or minimal direct financial cost to participating in the HPGC program as resources were provided to clubs without charge (e.g., posters printed by Gymnastics NSW, free coach education workshops) or clubs attained grants or donations to provision their activities (e.g., free fruit for club members). Indirect financial limitations were expressed by Gymnastics NSW staff and Participant-GOCc in relation to provisioning paid staff members who could dedicate their time to health promotion and thus sustain the program. Additionally, despite understanding the inconsistencies between selling unhealthy foods through the club and the overarching health-related focus of sport and physical activity, participants described dilemmas regarding the need to generate additional income for the club such as through chocolate fundraising drives.

### 4.3. Priorities and Timing

All participants described a conflict between recognizing the importance of promoting health through their settings and their ability to do so in light of other priorities reflecting their underlying core values. For example, Participant-MSCa summarized, “I don’t see [health promotion] as a negative…it’s just the [other] priorities for us.” All participants spoke of the prevailing priorities of their respective organizations, including a focus on service delivery, business and competitions or other events, indicating involvement in the project had not resulted, overall, in significant culture change placing health promotion among the core business of these organizations.

Business-related decisions influenced participants’ perceptions of the relative priority of health promotion for their organization. Gymnastics NSW’s Development Manager reported a positive shift of his perceptions and awareness of the organization’s role as a health-promoting setting but saw resourcing such initiatives in a sustainable way was a significant challenge for the future. Additionally, all club representatives felt maintaining a sustainable business model to be a priority and perceived the HPGC program could be a potential threat to achieving this.

Timing of the health promotion project was another prominent barrier. Timing was different to having limited time available. The former related to other events in the calendar year, school holidays, personal matters or organizational commitments taking precedence. The latter related to the theme of human capacity and workload discussed above. The point in the gymnastics calendar during which participating organizations were engaged in the intervention shaped the actions of both Gymnastics NSW and club staff. Overall, participants from both clubs and Gymnastics NSW described other tasks and matters as more urgent than health promotion. Their responses indicated it was not a ‘good time’ to do the project, a theme exemplified by statements such as, “There was so much more happening” (Participant-MSCa). Gymnastics NSW staff reiterated this, stating that other more pressing areas linked to external funds or investments made by Gymnastics NSW were prioritized, and providing the opinion on behalf of clubs the project was viewed as “a bit of a nice-to-do and not really a must-do like the day-to-day work” (Club Development Officer).

### 4.4. Project Involvement and Framework

#### 4.4.1. Voluntary Participation

The majority of club participants welcomed the voluntary nature of the HPGC program. Three participants (GOCb, MSCa and MSCb) were strongly averse to the idea of health promotion being a mandatory requirement for affiliation with Gymnastics NSW. Participants perceived the current compliance requirements were already sufficiently burdensome. Participant-GOCa was less resistant to a mandatory model but did stipulate any such requirements would need to be reasonable within the staffing capacity of any club and adequate support would be required from Gymnastics NSW. 

#### 4.4.2. Flexibility of Framework

The Club Development Officer perceived the flexibility or adaptability of the program framework positively as it provided clubs with “a list of things that they could do and the options and flexibility to let them pick and choose and decide what they’ve got time for and what they can really implement.” By contrast, the Development Manager reported the lack of a clear structure for the program, particularly regarding specification of time goals by which clubs would implement their selected activities, as a barrier to program ‘success’. 

From club participants’ perspectives, the agency available to select health promotion activities and set their own targets was a valued component of the program framework. Active participation in the action planning meetings and collaboration with Gymnastics NSW regarding the selection of program activities gave clubs a sense of ownership and ensured initiatives were relevant to their setting’s context. It also contributed to their self-efficacy regarding their abilities to implement health promotion activities within their existing capacity.

#### 4.4.3. Technical Support and Communication

Both the Development Manager and Club Development Officer unequivocally believed that the technical support they received from the researcher [A.C.] in framing the project and assisting with seeking health promotion resources and programs contributed positively to the project. The Development Manager reported that delivery of the program as a research project in collaboration with a tertiary education institution enhanced the validity of the program and secured a better response from clubs than what might have been the case had Gymnastics NSW introduced it as their own initiative. The researcher’s knowledge of existing health promotion resources and programs was also perceived as an asset to implementation and sustainability, resulting in “a resource library that we could share with clubs” (Club Development Officer), thus increasing the knowledge capacity of Gymnastics NSW staff.

Participants-GOCa and MSCb both reported feeling more positive toward face-to-face support from the researcher or Gymnastics NSW rather than support delivered via telephone or email. Participant-GOCa attributed this to the timeliness of receiving information from Gymnastics NSW, stating the use of telephone or email communication methods often resulted in delays. Participant-MSCb described communications with the researcher being a more logical association with health rather than the support offered by Gymnastics NSW, as exemplified by the statement, “Gymnastics NSW in my head is gymnastics, it’s not health” (Participant-MSCb). Participant-GOCc reported a positive impact of Gymnastics NSW staff visiting the club and explaining the project and concept of health promotion, stating “it didn’t sound as daunting an option”, but perceiving overall there was little scope for their club in the project, explaining, “the majority of [suggestions from Gymnastics NSW] we had already in place.” During the intervention phase, technical support extended to clubs by Gymnastics NSW was not accepted by Club-MSCa; however, Participant-MSCa reported favorable perceptions of the potential support available should the club engage in holistic health promotion in the future.

#### 4.4.4. Partnerships

External partnerships also influenced the outcomes of the HPGC program for two clubs and Gymnastics NSW. Club-MSCb was able to implement some strategies as the club manager forged a partnership between the club and the health promotion unit in the local health district department. This relationship enabled the club to become aware of and successfully apply for a funding grant to provision the free fruit for members and to utilize the services of smoking cessation workshops offered by the unit. It was possible for Club-GOCc to offer free fruit to members due to a partnership formed between the club and a local grocer, a partnership made more likely by a gymnast’s parent being employed at that grocery store.

Overall, Gymnastics NSW’s efforts to enlist the support of government departments at multiple time points throughout the project had limited success. During the early stages, the Development Manager brought the project to the attention of representatives from the NSW Office of Sport and NSW Department of Health. Throughout the intervention phase, the Club Development Officer contacted the health promotion unit from the local health district to investigate partnership opportunities that could be replicated in the state’s various gymnastic and health regions. However, the Development Manager reported these potential partners demonstrated a lack of engagement, citing subsequent limitations on the sustainability of the project in relation to finances:

*Our ability to get health promotion and the health promotion program into the limelight of Sport and Rec, our only funding partners, will continually be difficult because they’re not the body that sees it as necessary or a high priority to fund.*

(Development Manager)

While similarly recognizing that external partnerships had minimal influence during the current project, the Club Development Officer was confident the HPGC program contributed positively to the organization’s ability to develop necessary or beneficial connections in the future.

## 5. Discussion

Guided by organizational change theory, this study explored the implementation of the first participatory, whole-of-system, club-society development health-promoting settings approach to gymnastics. The potential for gymnastics as a setting to promote the wellbeing of all participants was the focus, rather than concentrating on a small proportion of elite athletes.

Various characteristics of organizational change theory were illustrated through exploration of the HPGC program. The program elicited incremental, developmental change [47]. In the duration of the HPGC program, the mode of change [48] was observable artefacts in the form of tangible health promotion activities, while organizational culture remained relatively unchanged, the potential reasons for which are discussed below. The level of change [49] was both within and across organizations as the changes at club level would not have occurred at the time of the project had it not been for Gymnastics NSW’s participation. Finally, the process of implementing the HPGC program was not consistent with the distinct and sequential stages described by linear models of organizational change processes [45,50], instead aligning more closely with complex models [54]. The change processes or patterns were diverse and were influenced by multiple internal and external factors and contexts.

As the study’s focus was organizational change, the unique type/s of organization and understanding how they function were important [27]. The gymnastics clubs in this study were predominantly small businesses, with associated organizational structures and cultures, such as having paid rather than volunteer staff. They existed within a hierarchical structure, with Gymnastics NSW being the state-level oversighting body that supported clubs to improve business operations and meet compliance requirements for affiliation. The HPGC program was responsive to these organizational characteristics. This contrasts with previous HPSC studies that used standardized intervention approaches within sports such as football, cricket and surf lifesaving [3]. Such sports (particularly for children) were predominantly run by volunteers [30], had different organizational cultures (participation vs. business) [27] and had different relationships with their parent organizations. 

The findings of this study support the importance of aligning strategies to the unique contexts of settings through a participatory approach [44,84]. Participating clubs selected strategies through the action planning process. Subsequent technical support was relevant to each discrete setting, enhancing the likelihood of adoption, implementation and sustainability of health promotion within participating organizations. The participatory approach of the program enabled it to be responsive to the needs, context and motivations of Gymnastics NSW and clubs. It also facilitated in-depth organizational involvement in program delivery, consistent with the principles of club-society development models of health-promoting settings [24]. 

The importance of organizational context in determining program implementation and outcomes [92] was confirmed by the varied nature and extent of influence of organizational factors (leadership; strategic, human and financial capacity; organizational priorities and timing; and elements of the program framework) in each club. In particular, the individual characteristics and beliefs of the leader within each gymnastics organization were integral across the course of adoption and implementation of the health promotion program, complementing the findings of previous studies such as those by Hoeber and Hoeber [93] and Olstad, Raine and McCargar [60]. Implementation of the program could have differed had there been other leaders in these positions at the time of the project.

Organizational capacity was crucial to the sustainability of the HPGC program in this study. Participants identified limited strategic, human and financial capacity as contributing to their inability to maintain or increase the extent of health promotion within their settings. This reflects findings of previous HPSC studies, such as conducted by Casey, Payne and Eime [57] and Olstad, Raine and McCargar [60], which reported organizations with smaller capacity face greater challenges when implementing sports-based health promotion innovations. This could be indicative of the need for increased support from multiple levels of sport and other sectors to ensure health promotion becomes an integral part of the core business of sport. Adequate capacity of community sports organizations to introduce and sustain changes depends on the number and nature (paid or volunteer) of staff and the agency of the organization to introduce changes to operations and the physical environment [93]. 

Pervasive organizational cultures were a significant barrier to generating and sustaining organizational change for health promotion in this study. Organizational leaders contribute significantly to the creation of organizational culture [52,94], exemplified in this study through their role as a gatekeeper to introducing innovations such as the HPGC program. The leader’s attitudes and perceptions of organizational priorities (e.g., business and competitions) considerably hindered any or timely implementation of health promotion activities and were barriers to the subsequent continuation, improvement or increase in health promotion activities in these settings. The findings that long-standing organizational cultures and ways of operating prove difficult to change, even when stakeholders’ perceptions may reportedly align with the values and potential positive outcomes of such an initiative, were consistent with those of previous studies [52,94]. Future studies need to incorporate comprehensive understanding of the cultures of participating organizations in order to maximize the impact and outcomes of organizational change.

Primary limitations of the current study are the small sample size of clubs participating in the program and the collection of data from only one representative of each club. It is not possible to assume all gymnastics clubs mirror the contextual molds of those involved in this study nor that they experience similar outcomes. However, principles of the study framework can enhance the appropriateness and relevance of health promotion initiatives to each unique club in the future. There was minimal stakeholder participation during post-intervention data collection processes within each organization, despite the study inviting the inclusion of multiple respondents from each organization. Notably, for clubs, this was characterized by the single perspective of the person considered most responsible for the whole club (e.g., owner and manager), and their innate perspectives will have shaped the data collected. The fact that organizational gatekeepers did not invite another representative from their club to participate in the post-intervention data collection may suggest a certain perspective on organizational change, the control they exerted over the club and the ingrained nature of communication and decision making in the club. Future research should endeavor to include the voices of multiple stakeholders with different roles from each organization (e.g., manager, parent, coach and gymnast) to minimize the potential biases of single respondents and to maximize the breadth of data collected.

We note that the data were collected and analyzed prior to the COVID-19 pandemic. While Australian gymnastics clubs, like all sport, experienced a short period of closure, clubs reopened within 6–9 months and were subject to government regulatory requirements to control the spread of infection (e.g., cleaning procedures and reduced class sizes). However, the potential for gymnastics (sport) clubs to be health-promoting settings is likely to remain as relevant at the time of publication as when the data were collected prior to the COVID-19 pandemic. This may be due, in part, to the presence of enduring organizational culture that often inhibits organizational change.

## 6. Conclusions

This research supports the potential of a low-cost, participatory, whole-of-system, club-society development health-promoting settings approach to gymnastics. Guided by organizational change theory, the study contributes to the body of evidence that identifies sports settings as positioned to create environments that promote health and wellbeing beyond participation in physical activity. The findings highlight the importance of embedding organizational change theory within similar initiatives, paying particular attention to the culture of participating organizations and key facilitators and barriers of the organizational change process. Despite some positive impacts, organizational culture (private enterprise and competition) inhibited adoption of health promotion as a core value in participating organizations. Sustained organizational change may result from professional regulatory requirements (e.g., accreditation and affiliation), policy direction and funding (for organizational change, not program delivery) from relevant government departments. To engender a cultural shift in sport such that it integrates health promotion within its core business, it will be important to take sustained action through participatory and whole-of-system approaches, draw on intersectoral partnerships and be responsive to the unique contexts of different sports organizations.

## Figures and Tables

**Table 1:** Characteristics of participating gymnastics organizations/clubs.

ID	Position	Purpose	Membership	Staffing	Summary of Organizational Change/Implemented Health Promotion Activities
Gymnastics NSW	State peak body	Promote, grow and develop gymnastics. Increase participation. Improve clubs’ business operations. Provide education for coaches and judges.	Approx. 190 gymnastics clubs. Approx. 56,477 registered gymnasts (2016) [76].	7 board members Chief Executive Officer 7 staff—Development Department 7 staff—Sport and Events Department Marketing Manager Accounts Supervisor	Allocated responsibility to specific state body staff. Healthy eating environment workshop for club staff at annual conference. Mental wellbeing online course.
**Club ID**	**Club type**	**Physical characteristics**	**Membership**	**Staffing (Coaches and other)**	**Summary of organizational change/implemented health promotion activities**
GOCa	Gymnastics-only club. Privately owned business. Competitive and recreational.	Permanently set-up. Large venue—industrial warehouse. Food preparation facilities. Drinking water available. Waiting area.	Large (approx. 250–300). Majority aged 18 months–14 years.	Approx. 20–25 casual paid coaches. 2 full-time paid management staff who were also coaches.	Coach education—healthy eating, mental wellbeing for gymnasts and first aid. Member wellbeing—health promotion posters and pamphlets (general wellbeing, healthy eating, injury prevention) and physical activity challenge.
GOCb	Gymnastics-only club. Privately owned. Predominantly competitive. Recreational for younger ages.	Permanently set-up. Small venue—shed. Drinking water available. Waiting area.	Moderate (approx. 100–150). Majority aged 5–20 years. Some 20–30 years.	Approx. 10 casual coaches: Paid or discounted lessons for gymnast-coaches. 1 full-time paid management staff who was also a coach.	Gymnast motivation and resilience.
GOCc	Gymnastics-only club. Incorporated association. Competitive and recreational.	Permanently set-up. Large venue. Located within multi-sport center, but separately operated. Canteen on-site. Drinking water available. Waiting area.	Large (at least 300). Majority aged 5–14 years.	Approx. 30–35 casual paid coaches. 1 paid management staff. Board members. 6 volunteers.	Member wellbeing—healthy eating (free fruit, posters), hydration.
MSCa	Multi-sport center. Leisure Center. Recreational	Set-up, pack-down. Large venue. Located and operated within leisure center. Canteen on-site. Vending machines. Drinking water available. Waiting area.	Moderate (approx. 150–200). Majority aged 18 months–14 years.	Approx. 10 casual paid coaches. Center manager. Front desk administrative staff. 5 full-time.	None.
MSCb	Multi-sport center. PCYC club. Predominantly recreational. Competitive for more advanced gymnasts.	Permanently set-up. Large venue. Food preparation facilities. Vending machines.	Moderate (approx. 100–150). Majority aged 18 months–14 years.	Approx. 10 casual coaches. Most volunteers. 1 paid full-time coach. Center manager (paid). Front desk administrative staff (mixture of paid and volunteer).	Member wellbeing—healthy eating (free fruit, healthy vending machine, healthy rewards). Coach education—mental wellbeing for gymnasts, smoking cessation.

NSW-New South Wales; GOC-Gymnastics only club; MSC-Multi-sport center; PCYC-Police Citizens Youth Club.

**Table 2:** Summary of facilitators and barriers to implementing the Health-Promoting Gymnastics Clubs program.

Organization ID	Leadership		Strategic Capacity		Human Capacity (Time and Workload)		Financial Capacity		Organizational Priorities and Timing		Voluntary Participation		Technical Support and Communication		Partnerships		Flexibility of Framework	
Gymnastics NSW	+	−	+		+	−		−	+				+			−		
Club-GOCa	+		+		+	−				−	+		+				+	
Club-GOCb	+	−	+	−	+	−		−		−	+					−	+	
Club-GOCc	+	−		−		−		−		−			+		+		+	
Club-MSCa		−		−		−		−		−								
Club-MSCb	+		+	−	+	−			+		+		+	−	+		+	

NSW-New South Wales; GOC-gymnastics only club; MSC-multi-sport center. + indicates the organization experienced that factor as a facilitator. − indicates the organization experienced that factor as a barrier. If a factor is marked as both facilitator and barrier for an organization, it is because that factor did exert both influences.

## Data Availability

The data presented in this study are available on request from the corresponding author. The data are not publicly available due to constraints within the approval granted by the ethics committee.

## Supplementary Materials

The following are available online at https://www.mdpi.com/article/10.3390/ijerph18136726/s1, File S1: Interview Question Guides.

## References

[1] World Health Organization (WHO) (1986). The Ottawa Charter for Health Promotion.

[2] Wilkinson R., Marmot M. (2003). Social Determinants of Health: The Solid Facts.

[3] Geidne S., Kokko S., Lane A., Ooms L., Vuillemin A., Seghers J., Koski P., Kudlacek M., Johnson S., Van Hoye A. (2019). Health Promotion Interventions in Sports Clubs: Can We Talk About a Setting-Based Approach? A Systematic Mapping Review. Health Educ. Behav..

[4] Geidne S., Quennerstedt M., Eriksson C. (2013). The youth sports club as a health-promoting setting: An integrative review of research. Scand. J. Public Heath.

[5] McFadyen T., Chai L.K., Wyse R., Kingsland M., Yoong S.L., Clinton-McHarg T., Bauman A., Wiggers J., Rissel C., Williams C.M. (2018). Strategies to improve the implementation of policies, practices or programmes in sporting organisations targeting poor diet, physical inactivity, obesity, risky alcohol use or tobacco use: A systematic review. BMJ Open.

[6] Eime R.M., Young J.A., Harvey J.T., Charity M.J., Payne W.R. (2013). A systematic review of the psychological and social benefits of participation in sport for children and adolescents: Informing development of a conceptual model of health through sport. Int. J. Behav. Nutr. Phys. Act..

[7] Warburton D.E.R., Nicol C.W., Bredin S.S.D. (2006). Health benefits of physical activity: The evidence. CMAJ.

[8] Tonts M. (2005). Competitive sport and social capital in rural Australia. J. Rural Stud..

[9] Nelson T.F., Stovitz S.D., Thomas M., LaVoi N.M., Bauer K.W., Neumark-Sztainer D. (2011). Do Youth Sports Prevent Pediatric Obesity? A Systematic Review and Commentary. Curr. Sports Med. Rep..

[10] Diehl K., Thiel A., Zipfel S., Mayer J., Litaker D.G., Schneider S. (2012). How Healthy is the Behavior of Young Athletes? A Systematic Literature Review and Meta-Analyses. J. Sports Sci. Med..

[11] Corti B., Holman C.D.J., Donovan R.J., Frizzell S.K., Carroll A.M. (1995). Using sponsorship to create healthy environments for sport, racing and arts venues in Western Australia. Health Promot. Int..

[12] Macniven R., Kelly B., King L. (2015). Unhealthy product sponsorship of Australian national and state sports organisations. Health Promot. J. Aust..

[13] Watson W.L., Brunner R., Wellard L., Hughes C. (2016). Sponsorship of junior sport development programs in Australia. Aust. N. Z. J. Public Health.

[14] Rosenberg M., Ferguson R. (2014). Maintaining relevance: An evaluation of health message sponsorship at Australian community sport and arts events. BMC Public Health.

[15] Australian Bureau of Statistics Children’s Participation in Cultural and Leisure Activities, Australia April 2012. http://www.abs.gov.au/AUSSTATS/abs@.nsf/productsbyCatalogue/0B14D86E14A1215ECA2569D70080031C.

[16] Australian Bureau of Statistics Involvement in Organised Sport and Physical Activity, Australia, April 2010. http://www.abs.gov.au/ausstats/abs@.nsf/mf/6285.0.

[17] Australian Bureau of Statistics Employment in Sport and Recreation, Australia, August 2011. http://www.abs.gov.au/ausstats/abs@.nsf/mf/4148.0.

[18] Australian Bureau of Statistics Sports and Physical Recreation: A Statistical Overview, Australia, 2012. http://www.abs.gov.au/ausstats/abs@.nsf/Products/3F9628655FD5552FCA257AD9000E2B33.

[19] Sport Australia Sport Australia: About. https://www.sportaus.gov.au/sportaus/about.

[20] Kelly B., King L., Bauman A.E., Baur L.A., Macniven R., Chapman K., Smith B. (2014). Identifying important and feasible policies and actions for health at community sports clubs: A consensus-generating approach. J. Sci. Med. Sport.

[21] Kokko S., Kannas L., Villberg J. (2006). The health promoting sports club in Finland—A challenge for the settings-based approach. Health Promot. Int..

[22] Kokko S. (2014). Guidelines for Youth Sports Clubs to Develop, Implement, and Assess Health Promotion Within Its Activities. Health Promot. Promot..

[23] Whitelaw S., Baxendale A., Bryce C., MacHardy L., Young I., Witney E. (2001). Settings’ based health promotion: A review. Health Promot. Int..

[24] Kokko S., Green L.W., Kannas L. (2014). A review of settings-based health promotion with applications to sports clubs. Health Promot. Int..

[25] Gold J., Hocking J., Hellard M. (2007). The feasibility of recruiting young men in rural areas from community football clubs for STI screening. Aust. N. Z. J. Public Health.

[26] Belski R., Staley K., Keenan S., Skiadopoulos A., Randle E., Donaldson A., O’Halloran P., Kappelides P., O’Neil S., Nicholson M. (2017). The impact of coaches providing healthy snacks at junior sport training. Aust. N. Z. J. Public Health.

[27] Casey M.M., Payne W.R., Eime R.M. (2009). Building the health promotion capacity of sport and recreation organisations: A case study of Regional Sports Assemblies. Manag. Leis..

[28] Casey M.M., Payne W.R., Brown S.J., Eime R.M. (2009). Engaging community sport and recreation organisations in population health interventions: Factors affecting the formation, implementation, and institutionalisation of partnerships efforts. Ann. Leis. Res..

[29] Casey M.M., Payne W.R., Eime R.M., Brown S.J. (2009). Sustaining health promotion programs within sport and recreation organisations. J. Sci. Med. Sport.

[30] Crisp B.R., Swerissen H. (2003). Critical processes for creating health-promoting sporting environments in Australia. Health Promot. Int..

[31] Dobbinson S.J., Hayman J.A., Livingston P.M. (2006). Prevalence of health promotion policies in sports clubs in Victoria, Australia. Health Promot. Int..

[32] Duff C., Munro G. (2007). Preventing Alcohol-Related Problems in Community Sports Clubs: The Good Sports Program. Subst. Use Misuse.

[33] Kingsland M., Wolfenden L., Tindall J., Rowland B., Sidey M., McElduff P., Wiggers J.H. (2015). Improving the implemen-tation of responsible alcohol management practices by community sporting clubs: A randomised controlled trial. Drug Alcohol Rev..

[34] Good Sports This Is Good Sports. http://goodsports.com.au/this-is-good-sports/.

[35] Geidne S., Quennerstedt M., Eriksson C. (2013). The implementation process of alcohol policies in eight Swedish football clubs. Health Educ..

[36] Drygas W., Ruszkowska J., Philpott M., Björkström O., Parker M., Ireland R., Roncarolo F., Tenconi M. (2013). Good practices and health policy analysis in European sports stadia: Results from the “Healthy Stadia” project. Health Promot. Int..

[37] Priest N., Armstrong R., Doyle J., Waters E. (2008). Policy interventions implemented through sporting organisations for promoting healthy behaviour change. Cochrane Database Syst. Rev..

[38] Priest N., Armstrong R., Doyle J., Waters E. (2008). Interventions implemented through sporting organisations for increasing participation in sport. Cochrane Database Syst. Rev..

[39] Laverack G., Labonte R. (2000). A planning framework for community empowerment goals within health promotion. Health Policy Plann..

[40] Dooris M. (2004). Joining up settings for health: A valuable investment for strategic partnerships?. Crit. Public Health.

[41] Dooris M. (2005). Healthy settings: Challenges to generating evidence of effectiveness. Health Promot. Int..

[42] Paton K., Sengupta S., Hassan L. (2005). Settings, systems and organization development: The Healthy Living and Working Model. Health Promot. Int..

[43] Jones G. (2007). Organizational Theory, Design, and Change.

[44] Al-Haddad S., Kotnour T. (2015). Integrating the organizational change literature: A model for successful change. J. Organ. Chang. Manag..

[45] Lewin K. (1951). Field Theory in Social Science.

[46] Batras D., Duff C., Smith B.J., Smith B. (2014). Organizational change theory: Implications for health promotion practice. Health Promot. Int..

[47] McNamara C. (2006). Clearing up the language about organizational change and development. Field Guide to Consulting and Organizational Development: A Collaborative and Systems Approach to Performance, Change and Learning.

[48] Meyer A.D., Brooks G.R., Goes J.B. (1990). Environmental jolts and industry revolutions: Organizational responses to discontinuous change. Strateg. Manag. J..

[49] Butterfoss F., Kegler M., Francisco V., Glanz K., Rimer B., Viswanath K. (2008). Mobilizing organizations for health promotion: Theories of organizational change. Health Behaviour and Health Education.

[50] Kaluzny A.D., Hernandez S.R. (1983). Review Article: Managing Change in Health Care Organizations. Med. Care Rev..

[51] Kotter J.P., Price D. (2009). Leading Change: Why Transformation Efforts Fail. Princ. Pract. Chang..

[52] Galpin T.J. (1996). The Human Side of Change: A Practical Guide to Organization Redesign.

[53] Taylor M.J., McNicholas C., Nicolay C., Darzi A., Bell D., Reed J.E. (2014). Systematic review of the application of the plan-do-study-act method to improve quality in healthcare. BMJ Qual. Saf..

[54] Styhre A. (2002). Non-linear change in organizations: Organization change management informed by complexity theory. Leadersh. Organ. Dev. J..

[55] Amagoh F. (2008). Perspectives on organizational change: Systems and complexity theories. Innov. J..

[56] Kremser W. (2010). Phases of school health promotion implementation through the lens of complexity theory: Lessons learnt from an Austrian case study. Health Promot. Int..

[57] Casey M.M., Payne W.R., Eime R.M. (2012). Organisational readiness and capacity building strategies of sporting organisations to promote health. Sport Manag. Rev..

[58] Casey M., Harvey J., Eime R.M., Payne W.R. (2012). Examining changes in the organisational capacity and sport-related health promotion policies and practices of State Sporting Organizations. Ann. Leis. Res..

[59] Olstad D.L., Downs S.M., Raine K.D., Berry T.R., McCargar L.J. (2011). Improving children’s nutrition environments: A survey of adoption and implementation of nutrition guidelines in recreational facilities. BMC Public Health.

[60] Olstad D.L., Raine K.D., McCargar L.J. (2012). Adopting and implementing nutrition guidelines in recreational facilities: Public and private sector roles. A multiple case study. BMC Public Health.

[61] Naylor P.-J., Olstad D.L., Therrien S. (2015). An Intervention to enhance the food environment in public recreation and sport settings: A natural experiment in British Columbia, Canada. Child. Obes..

[62] Olstad D.L., Lieffers J.R., Raine K., McCargar L.J. (2011). Implementing the Alberta Nutrition Guidelines for Children and Youth: In a Recreational Facility. Can. J. Diet. Pract. Res..

[63] Eime R.M., Payne W.R., Harvey J.T. (2008). Making sporting clubs healthy and welcoming environments: A strategy to in-crease participation. J. Sci. Med. Sport.

[64] Crisp B., Swerissen H., Duckett S.J. (2000). Four approaches to capacity building in health: Consequences for measurement and accountability. Health Promot. Int..

[65] Liberato S.C., Brimblecombe J., Ritchie J., Ferguson M., Coveney J. (2011). Measuring capacity building in communities: A review of the literature. BMC Public Health.

[66] Eriksson C., Geidne S., Larsson M., Pettersson C. (2011). A Research Strategy Case Study of Alcohol and Drug Prevention by Non-Governmental Organizations in Sweden 2003–2009. Subst. Abus. Treat. Prev. Policy.

[67] Naylor P.-J., Vander Wekken S., Trill D., Kirbyson A. (2010). Facilitating healthier food environments in public recreation facilities: Results of a pilot project in British Columbia, Canada. J. Park Recreat. Adm..

[68] Meganck J., Scheerder J., Thibaut E., Seghers J. (2015). Youth sports clubs’ potential as health-promoting setting: Profiles, motives and barriers. Health Educ. J..

[69] Naylor P.-J., Bridgewater L., Purcell M., Ostry A., Wekken S.V. (2010). Publically Funded Recreation Facilities: Obesogenic Environments for Children and Families?. Int. J. Environ. Res. Public Health.

[70] Thomas H.M., Irwin J. (2010). Food choices in recreation facilities: Operators’ and patrons’ perspectives. Can. J. Diet. Pract. Res..

[71] Young K., Kennedy V., Kingsland M., Sawyer A., Rowland B., Wiggers J., Wolfenden L. (2012). Healthy food and beverages in senior community football club canteens in New South Wales, Australia. Health Promot. J. Aust..

[72] Meganck J., Seghers J., Scheerder J. (2016). Exploring strategies to improve the health promotion orientation of Flemish sports clubs. Health Promot. Int..

[73] Skille E.Å. (2010). Competitiveness and health: The work of sport clubs as seen by sport clubs representatives—a Norwegian case study. Int. Rev. Sociol. Sport.

[74] Yin R.K. (2018). Case Study research and Applications: Design and Methods.

[75] Stake R. (2014). The multicase study. Multiple Case Study Analysis.

[76] Gymnastics NSW Gymnastics NSW 2016 Annual Report. http://www.gymnsw.org.au/NSW/About_Us/Publications/NSW/About/Publications.aspx.

[77] Creswell J.W., Hanson W.E., Plano Clark V.L., Morales A. (2007). Qualitative research designs: Selection and implementation. Couns. Psychol..

[78] Cassell C., Symon G. (2004). Essential Guide to Qualitative Methods in Organizational Research. Essential Guide to Qualitative Methods in Organizational Research.

[79] Green L.W., Raeburn J.M. (1988). Health promotion. What is it? What will it become?. Health Promot. Int..

[80] Kickbusch I. (2003). The Contribution of the World Health Organization to a New Public Health and Health Promotion. Am. J. Public Health.

[81] Potvin L., Jones C.M. (2011). Twenty-five Years After the Ottawa Charter: The Critical Role of Health Promotion for Public Health. Can. J. Public Health.

[82] Maben J., Clark J.M. (2006). Health promotion: A concept analysis. J. Adv. Nurs..

[83] Wise M., Signal L. (2000). Health promotion development in Australia and New Zealand. Health Promot. Int..

[84] Beets M.W., Weaver R.G., Turner-McGrievy G., Huberty J., Ward D.S., Freedman D.A., Saunders R., Pate R.R., Beighle A., Hutto B. (2014). Making healthy eating and physical activity policy practice: The design and overview of a group randomized controlled trial in afterschool programs. Contemp. Clin. Trials.

[85] NSW Government Make Healthy Normal. https://www.makehealthynormal.nsw.gov.au/home.

[86] Braun V., Clarke V. (2006). Using thematic analysis in psychology. Qual. Res. Psychol..

[87] Fereday J., Muir-Cochrane E. (2006). Demonstrating rigor using thematic analysis: A hybrid approach of inductive and deductive coding and theme development. Int. J. Qual. Meth..

[88] Mayring P. Qualitative Content Analysis: Theoretical Foundation, Basic Procedures and Software Solution. https://www.ssoar.info/ssoar/handle/document/39517.

[89] Shenton A.K. (2004). Strategies for ensuring trustworthiness in qualitative research projects. Educ. Inf..

[90] Cutler L., Bassett C. (2004). Ethnography. Qualitative Research in Health Care.

[91] Berger R. (2015). Now I see it, now I don’t: Researcher’s position and reflexivity in qualitative research. Qual. Res..

[92] Van Hoye A., Johnson S., Lemonnier F., Rostan F., Crochet L., Tezier B., Vuillemin A. (2021). Capitalization of Health Promotion Initiatives within French Sports Clubs. Int. J. Environ. Res. Public Health.

[93] Hoeber L., Hoeber O. (2012). Determinants of an innovation process: A case study of technoiogical innovation in a community sport organization. J. Sport Manag..

[94] Tierney W.G., Schein E.H. (2010). Organizational Culture and Leadership.

