# Supplementary material for: Building Public Health Capacity through Organizational Change in the Sport System: A Multiple-Case Study within Australian Gymnastics
Source: Int J Environ Res Public Health. 2021 Jun 22;18(13):6726. doi: 10.3390/ijerph18136726 (PMC8297274; doi:10.3390/ijerph18136726)
Supplement: Supplementary file 1 [file ijerph-18-06726-s001.zip › ijerph-1218481-supplementary.pdf]

Interview question guides

Gymnastics NSW staff

- Please can you tell me about Gymnastics NSW's experience of the Health Promoting Gymnastics Clubs (HPGC) program?

  Prompts:
  - What did you do?
  - How did it go?
  - How did you monitor how it went?
  - What helped?
  - What hindered?
  - Who was involved?
  - What was the response of staff to the program?
  - What was the response of clubs to the program?
  - What was the response from other stakeholders/partners to the program?
- I'm particularly interested in how organizations undergo and manage change. Can you tell me about a change that Gymnastics NSW had to manage in the past? What happened?
  - E.g. change to strategic direction
  - E.g. self-induced change
  - How did it go? What helped? What hindered?
- Describe any changes to Gymnastics NSW that have occurred as a result of the HPGC program.
  - Tangible activities implemented
  - Administrative changes, e.g., to resource allocation
  - Change to staff roles/ethos
  - Culture
  - Strategic focus
- Have there been any other changes in Gymnastics NSW that have happened over the 2 years? Can you describe them and what happened as a result?
- [Prompts regarding organizational change components, if not already discussed in early questions regarding barriers and facilitators to participation in the HPGC program]
  - What was the role of leadership in the program?
  - How did the HPGC program fit into your workload? (How much time did it require?)
  - Can you comment on the quality and suitability of resources provided to assist your club's participation in the program?
- How likely is it that your club will continue/start/increase health promotion activities?
- [Additional thoughts] Is there anything else you might like to add?

Club respondents

- Please can you tell me about your club's experience of the HPGC program?
  Prompts:
  - What did you do?
  - How did it go?
  - How did you monitor how it went?
  - What helped?
  - What hindered?
  - Who was involved?
  - What was the response of staff to activities?
  - What was the response of parents to activities?
  - What was the response of children to activities?
  - What was the response of the club to activities?
- I'm particularly interested in how clubs undergo and manage change. Can you tell me about a change that you/your club had to manage in the past? What happened?
  - E.g. change to accreditation/affiliation requirements.
  - E.g. self-induced change.
  - How did it go? What helped? What hindered?
  - How did you/your club go about meeting that requirement? What was your experience of it (ease, support, clarity, communication from Gymnastics NSW, cost, convenience)? What value do you perceive in it? What was your motivation? Did you/the club have to make additional changes to meet the new requirement?
- Describe any changes to your club that have occurred as a result of the HPGC program.
  - Tangible activities implemented
  - Administrative changes, e.g., to resource allocation
  - Change to staff roles/ethos
  - Culture
  - Strategic focus
- Have there been any other changes in your club that have happened over the last 2 years? Can you describe them and what happened as a result?
- [Prompts regarding organizational change components, if not already discussed in early questions regarding barriers and facilitators to participation in the HPGC program]
  - What was the role of leadership in the program?
  - How did the HPGC program fit into your workload? (How much time did it require?)
  - Can you comment on the quality and suitability of resources provided to assist your club's participation in the program?
  - Can you comment on the support your club received from Gymnastics NSW throughout the program?
- How likely is it that your club will continue/start/increase health promotion activities?
- [Additional thoughts] Is there anything else you might like to add?
